# An enhanced variant effect predictor based on a deep generative model and the Born-Again Networks

**DOI:** 10.1038/s41598-021-98693-3

**Published:** 2021-09-27

**Authors:** Ha Young Kim, Woosung Jeon, Dongsup Kim

**Affiliations:** Department of Bio and Brain Engineering, Korea Advanced Institute of Science and Technology, Daejeon, 34141 Republic of Korea

**Keywords:** Computational biology and bioinformatics, Machine learning, Protein analysis

## Abstract

The development of an accurate and reliable variant effect prediction tool is important for research in human genetic diseases. A large number of predictors have been developed towards this goal, yet many of these predictors suffer from the problem of data circularity. Here we present MTBAN (Mutation effect predictor using the Temporal convolutional network and the Born-Again Networks), a method for predicting the deleteriousness of variants. We apply a form of knowledge distillation technique known as the Born-Again Networks (BAN) to a previously developed deep autoregressive generative model, mutationTCN, to achieve an improved performance in variant effect prediction. As the model is fully unsupervised and trained only on the evolutionarily related sequences of a protein, it does not suffer from the problem of data circularity which is common across supervised predictors. When evaluated on a test dataset consisting of deleterious and benign human protein variants, MTBAN shows an outstanding predictive ability compared to other well-known variant effect predictors. We also offer a user-friendly web server to predict variant effects using MTBAN, freely accessible at http://mtban.kaist.ac.kr. To our knowledge, MTBAN is the first variant effect prediction tool based on a deep generative model that provides a user-friendly web server for the prediction of deleteriousness of variants.

## Introduction

While recent sequencing technologies have resulted in a tremendous amount of sequence variant data, the identification of deleterious variants is still a difficult problem. Development of a reliable computational tool to predict the effects of sequence variants would aid in the treatment of many human genetic diseases. To achieve this goal, many predictors have been developed based on different approaches. Among these methods, supervised methods learn from labelled variant data consisting of known deleterious and benign variants, and many of them show good predictive ability. However, many supervised methods face the problem of data circularity, which can be divided into two types according to Grimm et al*.*^1^ The *type I circularity* arises due to the overlap between training data and test data. The *type II circularity* occurs when all variants in a given gene are labelled either all deleterious or all benign, which results in the model predicting the same label for all variants in that gene. Previous studies^1–3^ have suggested that this problem of data circularity can result in an inflation of the reported performances of many supervised predictors. On the other hand, unsupervised methods do not learn from labelled variant data and learn solely from the evolutionary information contained in multiple sequence alignments. A recent study which carried out an extensive comparison of variant effect predictors claimed that a class of unsupervised models, namely the deep generative model, is a promising area of research for variant effect prediction^3^.

Here, we introduce MTBAN (Mutation effect predictor using the Temporal convolutional network and the Born-Again Networks), an enhanced method to predict the deleteriousness of single amino acid variants. We previously developed a method called mutationTCN^4^ based on a deep autoregressive generative model, and showed that it demonstrates state-of-the-art performances on the prediction of functional effects of variants. In this work, we apply a knowledge distillation technique called the Born-Again Networks (BAN)^5^ to the mutationTCN model and develop an improved model called MTBAN. In machine learning, knowledge distillation is a process involving the transfer of knowledge learned from one machine learning model to another. Using the Born-Again Networks allows the student network to achieve an improved predictive power compared to the teacher network. When evaluated on human variant datasets with deleterious and benign variants, MTBAN shows superior predictive performances compared to other variant effect predictors. Our model is fully unsupervised and is not dependent on labelled data for training. This gives the model advantage over supervised predictors, for which data circularity is an inherent problem. We also offer a freely accessible web server for using MTBAN for variant effect prediction.

## Methods

### MTBAN model

**Figure 1:**
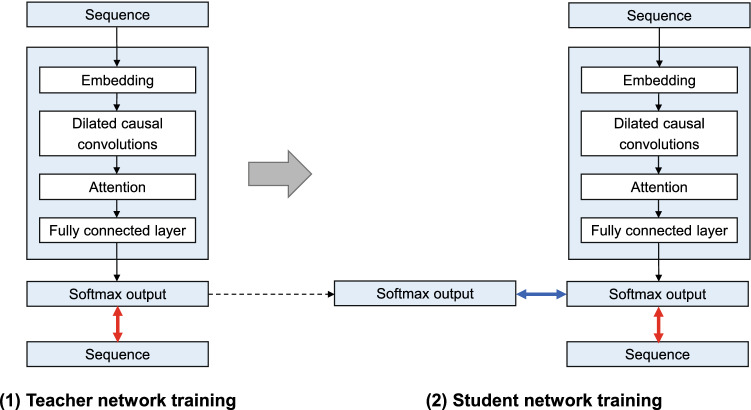
MTBAN model structure. We implemented BAN with mutationTCN as both the teacher and the student network. In the first step, only the teacher network is trained, with the loss function being the label loss (red arrow), which refers to the cross entropy loss between the input sequence and the softmax output distribution of the teacher network. In the second step, only the student network is trained, with the loss being the sum of the label loss (red arrow) and the teacher loss (blue arrow). Here, the label loss refers to the cross entropy loss between the input sequence and the softmax output of the student network. The teacher loss refers to the cross entropy loss between the softmax output of the student network and the “softened” output distribution of the teacher network.

We previously developed a deep autoregressive generative model for variant effect prediction, called mutationTCN^4^. For each protein variant, the model is trained on the multiple sequence alignment of the corresponding protein. As it is a generative model, it is trained by maximizing the likelihood of the training data, which is equivalent to minimizing the negative log likelihood between the input sequence and the predicted output. After training, the model can predict the probability of observing a given protein sequence under the parameters of the trained model. The deep autoregressive generative model is implemented using the temporal convolutional network architecture^6^. Each sequence from the input multiple sequence alignment is encoded by representing each amino acid in the sequence as a distinct integer. The input is passed through an embedding layer, followed by a series of dilated causal convolution layers, an attention layer, and a fully connected layer (Fig. 1). We showed that this model can effectively capture information from evolutionarily related sequences and use this information to predict the functional effects of variations in a sequence^4^.

MTBAN combines this model with a knowledge distillation technique in machine learning, known as the Born-Again Networks (BAN)^5^. Knowledge distillation is a process of transferring the knowledge from one machine learning model to another^7^. In this scheme, the former is referred to as the “teacher” model and the latter is referred to as the “student” model. Typically, knowledge is transferred from a larger model to a smaller model, which allows for the reduction of model size while maintaining similar predictive power as the original model. In the setting of BAN, the student network is of the same capacity as the teacher network, which enables the student network to outperform the teacher network^5^. We found that the BAN framework in which both the teacher and the student network is implemented with mutationTCN outperforms the original mutationTCN model.

The model structure of MTBAN is shown in Fig. 1. In the first step, only the teacher network is trained, with the loss function being the *label loss*, which refers to the cross entropy loss between the input sequence and the softmax output distribution of the teacher network. In the next step, only the student network is trained, with the loss being the sum of the *label loss* and the *teacher loss*. Here, the *label loss* refers to the cross entropy loss between the input sequence and the softmax output of the student network. The *teacher loss* refers to the cross entropy loss between the softmax output of the student network and the softmax output of the teacher network. The softmax output distribution ${p}_{i}$ of the teacher network can be expressed as follows:

$${p}_{i}=\frac{\mathrm{exp}(\frac{{z}_{i}}{T})}{\sum_{j}\mathrm{exp}(\frac{{z}_{j}}{T})}$$

where ${z}_{i}$ is the logit computed for each class and $T$ is the temperature parameter, which is typically set to 1^7^. Using higher temperatures leads to more “softened” output distributions. According to Hinton et al.^7^, these softened output distributions contain the “dark knowledge,” which is the hidden knowledge learned by the teacher network. In BAN, the transfer of this “dark knowledge” from the teacher to the student contributes to the improved performance of the student network. In our implementation, we used a temperature of 4. We trained both teacher and student networks for 500,000 iterations using the mini-batches with the size of 128. For both teacher and student networks, the learning rate is set to 0.001 when the number of training iterations is smaller than 3000, and 0.0001 when it is greater than 3000.

We computed the predictions of MTBAN for a total of 1605 human protein alignments provided by Hopf et al*.*^8^ These pre-computed predictions on the Hopf dataset were used for evaluating the model on the test set. According to Hopf et al*.*, their alignment generation protocol involves multiple iterations of profile HMM homology search in an attempt to ensure that there are enough sequences in the alignment and that the alignment coverage of the target protein sequence domain is sufficient^8^. This allows us to obtain an alignment that contains as much evolutionary information as possible.

### Model outputs

For a given variant, the model outputs the log probability score, the z-score, the probability of deleteriousness, and the predicted label. First, the log probability score is given by the following:

$$\mathrm{log}\frac{p({x}^{mutant}|\theta )}{p({x}^{wild {\text{-}} type}|\theta )}$$

where $p({x}^{mutant}|\theta )$ and $p({x}^{wild {\text{-}} type}|\theta )$ are the probability assigned to the mutant sequence and the wild-type sequence, respectively, by the generative model with parameters $\theta$. The log probability score is easily computed from the loss function, as the model loss function is the negative log likelihood^4^. The smaller the score, the more likely the variant has a deleterious effect. Second, the z-score is computed by normalizing the distribution of log probability scores for all possible missense variants against the target protein sequence. This normalization process is done due to the variations in the score distributions across different proteins. Third, the probability of deleteriousness for each variant, ranging from 0 to 1, is computed. This is determined from the set of variants in the Humsavar database (release 03/2020)^9^ which overlap with our pre-computed model predictions for the Hopf dataset, which are 1221 deleterious and 1221 benign variants. We obtained the z-score distribution for this set of variants, divided the distribution into equal-length z-score intervals, and calculated the proportion of deleterious variants in each z-score interval. Finally, using the same z-score intervals, we determined a z-score cutoff which maximizes the classification accuracy (Supplementary Fig. S1). This cutoff is used to assign a predicted label, either deleterious or benign, to a given variant.

### Evaluation datasets

To evaluate the ability of the model to classify human protein variants as deleterious or benign, we created a test dataset by combining the variants from the datasets used by Grimm et al.^1^ and Mahmood et al.^2^ Details regarding the datasets can be found in Table 1. We used the HumVar dataset from Grimm et al., which contains human protein variants that are known to be disease-causing or neutral^1^. Also, we used the UniFun, BRCA1-DMS, and TP53-TA datasets from Mahmood et al., which contain deleterious and benign protein variants determined from direct in vitro functional assays, such as the deep mutational scanning experiment^2^. Mahmood et al. pointed out that commonly used disease-related variant datasets often overlap with the training data used by supervised predictors^2^. Because of this reason, they created the functionally determined variant datasets in order to avoid the problem of data circularity and establish an independent test set for benchmarking^2^. Another study^3^ also supports this claim and uses the data from deep mutational scanning experiments to benchmark a large number of variant effect predictors. Also, it is reported that the Critical Assessment of Genome Interpretation (CAGI), which aims to perform an unbiased assessment of variant effect predictors, uses data from deep mutational scanning experiments as part of their benchmark dataset^10^. Therefore, we use the functionally determined variant data from Mahmood et al*.* in addition to the disease-related variant data for comparing MTBAN with other predictors.

**Table 1:** Test datasets used and the number of deleterious and benign variants for each dataset used for evaluation.

References	Dataset	Description	ND	NB
Grimm et al.^1^	HumVar	Disease-causing mutations from UniProtKB and common single nucleotide polymorphisms with major allele frequency > 1%^1^	1230	1230
	Total		1230	1230
Mahmood et al.^2^	UniFun	Deleterious and benign variants in UniProt which are derived from functional assays^2^	25	25
	BRCA1-DMS	Deleterious and benign variants derived from deep mutational scanning experiment measuring homology-directed DNA repair and tumor suppression activity^2^	41	41
	TP53-TA	Deleterious and benign variants derived from transactivation assay^2^	413	413
	Total		479	479
Total			1709	1709

ND stands for the number of deleterious variants, and NB stands for the number of benign variants.

We compared the performance of our model with mutationTCN and other commonly used variant effect predictors, SIFT^11^, PolyPhen-2^12^, MutationAssessor^13^, fathmm-MKL^14^, MPC^15^, GenoCanyon^16^, phastCons^17^, DANN^18^, GERP++^19^, and phyloP^20^. The predictions of the commonly used predictors on the test dataset were obtained from dbNSFP^21^ via the Ensembl variant effect predictor^22^. Since the score cutoffs for phyloP, DANN, phastCons, GERP++, MPC, and GenoCanyon were not provided by dbNSFP, we computed the cutoffs for each predictor using the Humsavar database (release 03/2021) as described in “Methods” section.

We found variants among the datasets from Grimm et al*.* and Mahmood et al*.* for which MTBAN predictions exist in the pre-computed Hopf dataset, and used those variants for comparison with other methods. Since the number of deleterious variants was significantly larger than that of benign variants, we randomly selected variants from the deleterious variant data to match the data size of the deleterious variants and the benign variants. This resulted in a balanced test set consisting of 1709 deleterious and 1709 benign variants in total.

### Evaluation criteria

The following metrics were used for evaluating the classification ability of the variant effect predictors: ROC-AUC (Receiver Operating Characteristic Area Under Curve), PR-AUC (Precision-Recall Area Under Curve), accuracy, Matthews Correlation Coefficient (MCC), precision, specificity, sensitivity, F-score, and Negative Predictive Value (NPV). For MTBAN, ROC-AUC and PR-AUC were calculated using z-scores, and other evaluation metrics were calculated using the predicted label. The following equations were used for computing the evaluation metrics:

$$\mathrm{Accuracy}=\frac{TP+TN}{TP+FN+TN+FP}$$

$$\text{Matthews} \; \text{Correlation} \; \text{Coefficient} \; (\mathrm{MCC})=\frac{TP\times TN-FP\times FN}{\sqrt{(TP+FP)\times (TP+FN)\times (TN+FP)\times (TN+FN)}}$$

$$\mathrm{Precision}=\frac{TP}{TP+FP}$$

$$\mathrm{Specificity}=\frac{TN}{FP+TN}$$

$$\mathrm{Sensitivity } \; (\mathrm{Recall})=\frac{TP}{TP+FN}$$

$$\mathrm{F}\text{-}\mathrm{score}=2\frac{Precision\times Recall}{Precision+Recall}$$

$$\text{Negative} \; \text{Predictive} \; \text{Value} \; (\mathrm{NPV})=\frac{TN}{TN+FN}$$

where TP, TN, FP, and FN are the number of true positives, true negatives, false positives, and false negatives, respectively.

## Results

### Evaluation on human protein variant datasets

**Figure 2:**
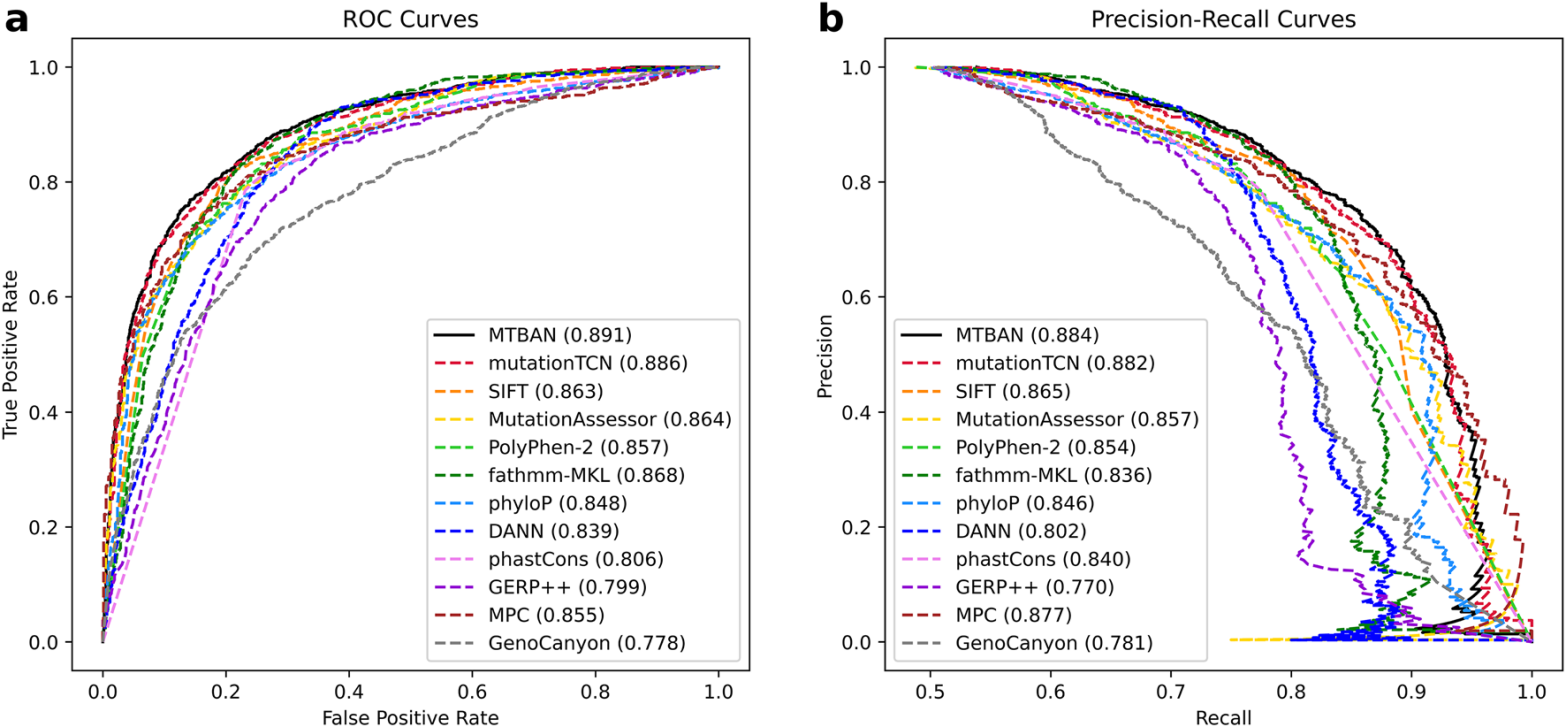
ROC Curves and Precision-Recall Curves for MTBAN and other predictors on the test dataset. (**a**) MTBAN achieved a ROC-AUC (Receiver Operating Characteristic Area Under Curve) of 0.883, which is the highest among 12 variant effect predictors. (**b**) MTBAN achieved a PR-AUC (Precision-Recall Area Under Curve) of 0.878, outperforming all other variant effect predictors.

We assessed MTBAN and other variant effect predictors on the task of classifying human protein variants as deleterious or benign. As described in “Methods” section, our test dataset combines the disease-associated variants from Grimm et al*.*^1^ and functionally determined variants from Mahmood et al.^2^, resulting in a total of 1709 deleterious and 1709 benign variants. When compared with 11 other variant effect predictors in terms of ROC-AUC and PR-AUC, our model outperformed all other predictors, achieving a ROC-AUC of 0.883 and a PR-AUC of 0.878 (Fig. 2, Table 2). Even though our model is fully unsupervised, its predictive ability outperforms the supervised predictors including PolyPhen-2, whose training dataset has overlapping variants with the dataset from Grimm et al.^1^ Also, MTBAN achieved the highest accuracy, MCC, and F-score among all compared variant effect predictors. In addition, our model demonstrates a good balance between specificity and sensitivity, unlike fathmm-MKL or phyloP which demonstrate good performance in only one of the two measures.

**Table 2:** Performances of MTBAN and other predictors on the test dataset consisting of 1709 deleterious and 1709 benign variants.

Predictor	ROC-AUC	PR-AUC	Accuracy	MCC	Precision	Specificity	Sensitivity	F-score	NPV
MTBAN	**0.883**	**0.878**	**0.787**	**0.585**	0.739	0.686	0.887	**0.806**	0.859
mutationTCN	0.873	0.87	0.763	0.548	0.706	0.624	0.902	0.792	0.865
SIFT	0.856	0.861	0.77	0.55	0.728	0.671	0.868	0.792	0.833
MutationAssessor	0.855	0.849	0.763	0.535	0.722	0.686	0.843	0.778	0.819
PolyPhen-2	0.853	0.856	0.759	0.537	0.703	0.637	0.885	0.783	0.851
fathmm-MKL	0.844	0.812	0.743	0.518	0.681	0.567	**0.918**	0.782	**0.873**
phyloP^a^	0.836	0.838	0.753	0.532	**0.865**	**0.905**	0.602	0.71	0.693
DANN	0.814	0.775	0.753	0.51	0.722	0.68	0.825	0.77	0.794
phastCons^b^	0.789	0.829	0.749	0.506	0.711	0.657	0.84	0.77	0.803
GERP++	0.778	0.74	0.714	0.435	0.757	0.795	0.635	0.69	0.684
MPC	0.772	0.762	0.68	0.369	0.73	0.772	0.591	0.653	0.644
GenoCanyon	0.742	0.748	0.657	0.323	0.626	0.53	0.783	0.696	0.708

Since the score cutoffs for phyloP, DANN, phastCons, GERP++, MPC, and GenoCanyon were not provided by dbNSFP, we computed the cutoffs for each predictor using the Humsavar database (release 03/2021) as described in “Methods” section. The highest values for each evaluation metric are indicated in bold.

ROC-AUC, Receiver Operating Characteristic Area Under Curve; PR-AUC, Precision-Recall Area Under Curve; MCC, Matthews Correlation Coefficient; NPV, Negative Predictive Value.

^a^PhyloP100way_vertebrate from dbNSFP.

^b^PhastCons100way_vertebrate from dbNSFP.

In addition, we conducted further assessment using only the disease-associated variant data from Grimm et al*.*^1^, and using only the functionally determined variant data from Mahmood et al.^2^ When tested on the data from Grimm et al*.* consisting of 1230 deleterious and 1230 benign variants, our model achieved the highest ROC-AUC, PR-AUC, accuracy, MCC, and F-score (Supplementary Table S1). Also, when tested on the data from Mahmood et al. consisting of 479 deleterious and 479 benign variants, our model achieved the highest ROC-AUC, accuracy, MCC, and F-score (Supplementary Table S2). Overall, MTBAN shows an outstanding classification ability in both disease-associated variant data and functional assay-derived variant data.

### Web server

We offer a user-friendly web server which predicts variant effects using MTBAN (Supplementary Fig. S2). The server takes in as input a protein UniProt accession and a list of amino acid variants. Upon receiving input, it determines the target protein sequence region, and checks if pre-computed predictions exist for the given variants. If they exist, the server immediately returns predictions to the user. Otherwise, it checks if a multiple sequence alignment of the target protein sequence region is present in the database. If an alignment is present, it uses that alignment for subsequent computations. If an alignment is not present, it generates one using a profile HMM homology search tool^23^ and saves it in the database. During the computation, alignment columns that have more than 30% gaps are dropped. If some of the input variants belong to these un-aligned columns in the alignment, those variants are excluded from prediction and are indicated in the results. The next step is the computation of sequence weights, based on the similarity of sequences in the alignment. This step is included to reduce any sequence bias present in the multiple sequence alignment^4^. Afterwards, the prediction model is trained, and the server returns predictions to the user. After job processing, the predictions are saved so that the server can immediately return the results when the same set of mutations are later submitted as input. In the web server implementation, due to time constraints, the MTBAN teacher network and student network are both trained for 200,000 iterations, with learning rate 0.001.

## Discussion

Here, we have introduced MTBAN, an improved method for predicting the deleteriousness of single amino acid variants. As demonstrated in our previous work^4^, the deep autoregressive generative model is a powerful tool for learning the distribution underlying the evolutionarily related sequences of a protein and predicting the effects of variations in a sequence. Combining the deep autoregressive generative model with a knowledge distillation method known as the Born-Again Networks (BAN) further improves the predictive power of the model, by transferring the knowledge learned by the model to the second model of the same capacity. We conducted an assessment using the test set combining the disease-related variants from Grimm et al*.*^1^ and the functionally determined variants from Mahmood et al*.*^2^, and further assessment using each of the two variant sets. In all cases, MTBAN consistently shows outstanding predictive ability compared to other prediction tools. The results indicate that MTBAN is a reliable method for predicting the deleteriousness of human protein variants.

Previous works^1–3^ have pointed out concerns regarding the problem of data circularity in many supervised predictors, which can lead to an inflation of the reported performances of these tools. Due to the fully unsupervised nature of MTBAN, it is not hindered by the problem of data circularity and can be considered to have higher generality compared to supervised models. Moreover, while we only considered human protein variants in this work, it is possible to predict the effects of protein variants in any other species if a multiple sequence alignment is available.

As previously mentioned, the BAN involves the transfer of the “dark knowledge” hidden in the softened output distribution of the teacher network to the student network. We speculate that due to the large size and the high complexity of the training set used in this study, the student equipped with the teacher’s knowledge can better model the distribution of the training data, compared to the teacher alone. In other scenarios where the model is of high capacity and the training data is limited in size, the student network may possibly perform worse due to overfitting.

One potential limitation of MTBAN and mutationTCN is that they can only make predictions for variants which correspond to the conserved positions in the multiple sequence alignment of a protein. However, when we analyzed all of the 9935 human protein multiple sequence alignments in the Hopf dataset, approximately 88% of the target sequences were conserved, which is a considerably large proportion. Another potential limitation of MTBAN is that the training time is longer compared to mutationTCN alone for prediction. Although MTBAN takes a longer time to train, it shows a higher predictive performance compared to the previous model.

The results of our work show that the deep generative model is a powerful tool for predicting the effects of sequence variations. We expect that deep generative models will continue to play an important role in discovering the effects of genetic variants. In addition, to our knowledge, MTBAN is the first variant effect prediction tool based on a deep generative model that provides a user-friendly web server for the prediction of deleteriousness of variants. This method is expected to be a useful tool for the prioritization and identification of variants involved in human genetic diseases.

## Supplementary Information

Supplementary Information.

## Data Availability

The datasets generated during and/or analyzed during the current study are available at https://github.com/ha01994/MTBAN.
