# Supplementary material for: An enhanced variant effect predictor based on a deep generative model and the Born-Again Networks
Source: Sci Rep. 2021 Sep 27;11:19127. doi: 10.1038/s41598-021-98693-3 (PMC8476491; doi:10.1038/s41598-021-98693-3)

**Supplementary Information:**

**An enhanced variant effect predictor based on a deep generative model and the Born-Again Networks**

**Ha Young Kim^1^, Woosung Jeon^1^ and Dongsup Kim^1,*^**

^1^Department of Bio and Brain Engineering, Korea Advanced Institute of Science and Technology, Daejeon 34141, Republic of Korea

*Corresponding author ([kds@kaist.ac.kr](mailto:kds@kaist.ac.kr))

**Supplementary Table S1. Performances of MTBAN and other predictors on the HumVar dataset from Grimm *et al*. consisting of total 1,230 deleterious and 1,230 benign variants.** Since the score cutoffs for phyloP, DANN, phastCons, GERP++, MPC, and GenoCanyon were not provided by dbNSFP, we computed the cutoffs for each predictor using the Humsavar database (release 03/2021) as described in the Methods section. The highest values for each evaluation metric are indicated in bold.

| Predictor | ROC-AUC | PR-AUC | Accuracy | MCC | Precision | Specificity | Sensitivity | F-score | NPV |
| --- | --- | --- | --- | --- | --- | --- | --- | --- | --- |
| MTBAN | **0.891** | **0.884** | **0.8** | **0.609** | 0.757 | 0.715 | 0.885 | **0.816** | 0.861 |
| mutationTCN | 0.886 | 0.882 | 0.788 | 0.587 | 0.74 | 0.689 | 0.887 | 0.807 | 0.859 |
| SIFT | 0.863 | 0.865 | 0.792 | 0.586 | 0.768 | 0.74 | 0.843 | 0.804 | 0.822 |
| MutationAssessor | 0.864 | 0.857 | 0.774 | 0.551 | 0.748 | 0.74 | 0.81 | 0.778 | 0.804 |
| PolyPhen-2 | 0.857 | 0.854 | 0.776 | 0.563 | 0.727 | 0.69 | 0.866 | 0.79 | 0.843 |
| fathmm-MKL | 0.868 | 0.836 | 0.777 | 0.576 | 0.718 | 0.636 | **0.916** | 0.805 | **0.883** |
| phyloP^a^ | 0.848 | 0.846 | 0.757 | 0.543 | **0.878** | **0.916** | 0.6 | 0.713 | 0.694 |
| DANN | 0.839 | 0.802 | 0.777 | 0.558 | 0.75 | 0.718 | 0.836 | 0.79 | 0.812 |
| phastCons^b^ | 0.806 | 0.84 | 0.761 | 0.527 | 0.726 | 0.681 | 0.84 | 0.779 | 0.808 |
| GERP++ | 0.799 | 0.77 | 0.731 | 0.467 | 0.769 | 0.798 | 0.665 | 0.713 | 0.702 |
| MPC | 0.855 | 0.877 | 0.775 | 0.571 | 0.872 | 0.895 | 0.662 | 0.753 | 0.712 |
| GenoCanyon | 0.778 | 0.781 | 0.657 | 0.34 | 0.614 | 0.459 | 0.854 | 0.714 | 0.756 |

ROC-AUC, Receiver Operating Characteristic Area Under Curve; PR-AUC, Precision-Recall Area Under Curve; MCC, Matthews Correlation Coefficient; NPV, Negative Predictive Value

^a^phyloP100way_vertebrate from dbNSFP.

^b^phastCons100way_vertebrate from dbNSFP.

**Supplementary Table S2. Performances of MTBAN and other predictors on the UniFun, BRCA1-DMS, and TP53-TA datasets from Mahmood *et al*. consisting of total 479 deleterious and 479 benign variants.** Since the score cutoffs for phyloP, DANN, phastCons, GERP++, MPC, and GenoCanyon were not provided by dbNSFP, we computed the cutoffs for each predictor using the Humsavar database (release 03/2021) as described in the Methods section. The highest values for each evaluation metric are indicated in bold.

| Predictor | ROC-AUC | PR-AUC | Accuracy | MCC | Precision | Specificity | Sensitivity | F-score | NPV |
| --- | --- | --- | --- | --- | --- | --- | --- | --- | --- |
| MTBAN | **0.863** | 0.865 | **0.753** | **0.527** | 0.697 | 0.612 | 0.894 | **0.783** | 0.852 |
| mutationTCN | 0.845 | 0.844 | 0.699 | 0.456 | 0.634 | 0.457 | **0.942** | 0.758 | **0.887** |
| SIFT | 0.86 | **0.871** | 0.722 | 0.485 | 0.659 | 0.522 | 0.923 | 0.769 | 0.871 |
| MutationAssessor | 0.851 | 0.83 | 0.743 | 0.513 | 0.686 | 0.584 | 0.902 | 0.779 | 0.855 |
| PolyPhen-2 | 0.854 | 0.864 | 0.726 | 0.491 | 0.663 | 0.531 | 0.921 | 0.771 | 0.87 |
| fathmm-MKL | 0.816 | 0.802 | 0.657 | 0.37 | 0.602 | 0.39 | 0.923 | 0.729 | 0.835 |
| phyloP^a^ | 0.809 | 0.823 | 0.743 | 0.505 | **0.832** | **0.877** | 0.61 | 0.704 | 0.692 |
| DANN | 0.734 | 0.676 | 0.69 | 0.389 | 0.656 | 0.582 | 0.797 | 0.72 | 0.742 |
| phastCons^b^ | 0.747 | 0.805 | 0.719 | 0.452 | 0.676 | 0.597 | 0.841 | 0.75 | 0.79 |
| GERP++ | 0.721 | 0.643 | 0.672 | 0.354 | 0.724 | 0.787 | 0.557 | 0.63 | 0.64 |
| MPC | 0.628 | 0.612 | 0.53 | 0.061 | 0.534 | 0.585 | 0.476 | 0.503 | 0.527 |
| GenoCanyon | 0.677 | 0.662 | 0.656 | 0.313 | 0.674 | 0.71 | 0.601 | 0.636 | 0.64 |

ROC-AUC, Receiver Operating Characteristic Area Under Curve; PR-AUC, Precision-Recall Area Under Curve; MCC, Matthews Correlation Coefficient; NPV, Negative Predictive Value

^a^phyloP100way_vertebrate from dbNSFP.

^b^phastCons100way_vertebrate from dbNSFP.

**Supplementary Figure S1. Distribution of MTBAN predictions (z-scores) for 1,221 deleterious and 1,221 benign protein variants obtained from the Humsavar database (release 03/2020).** From this z-score distribution, we divided the distribution into equal-length z-score intervals, calculated the proportion of deleterious variants in each z-score interval, and use this information to predict the probability of deleteriousness for a given variant. Also, from the same z-score intervals, we classify a variant as deleterious or benign based on a z-score cutoff (0.647) that maximizes the classification accuracy.


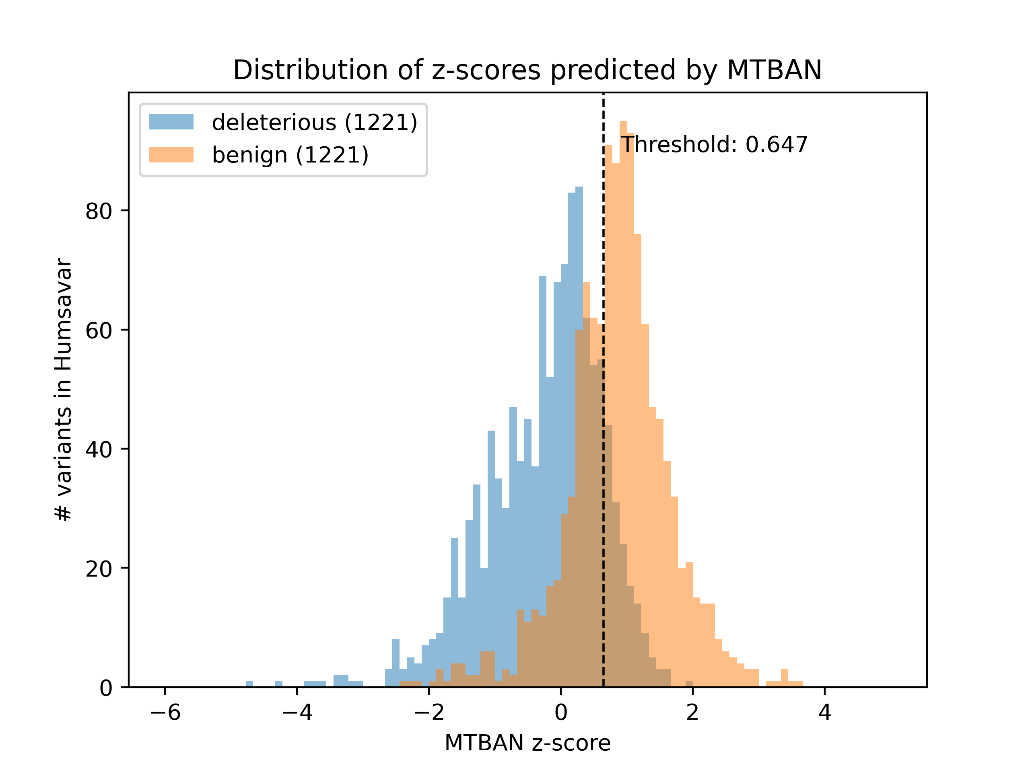


**Supplementary Figure S2. MTBAN web server workflow.** The web server takes in protein UniProt accession and a set of amino acid variants as input. First, it checks if pre-computed predictions exist and can be returned immediately to the user. If predictions do not exist, it checks if a sequence alignment of the target protein sequence is present in the database. If an alignment is present, it uses the alignment to train model; if not, it builds an alignment first. Subsequently, sequence weights are calculated, and the prediction model is trained. After model training, the predictions are returned to the user.


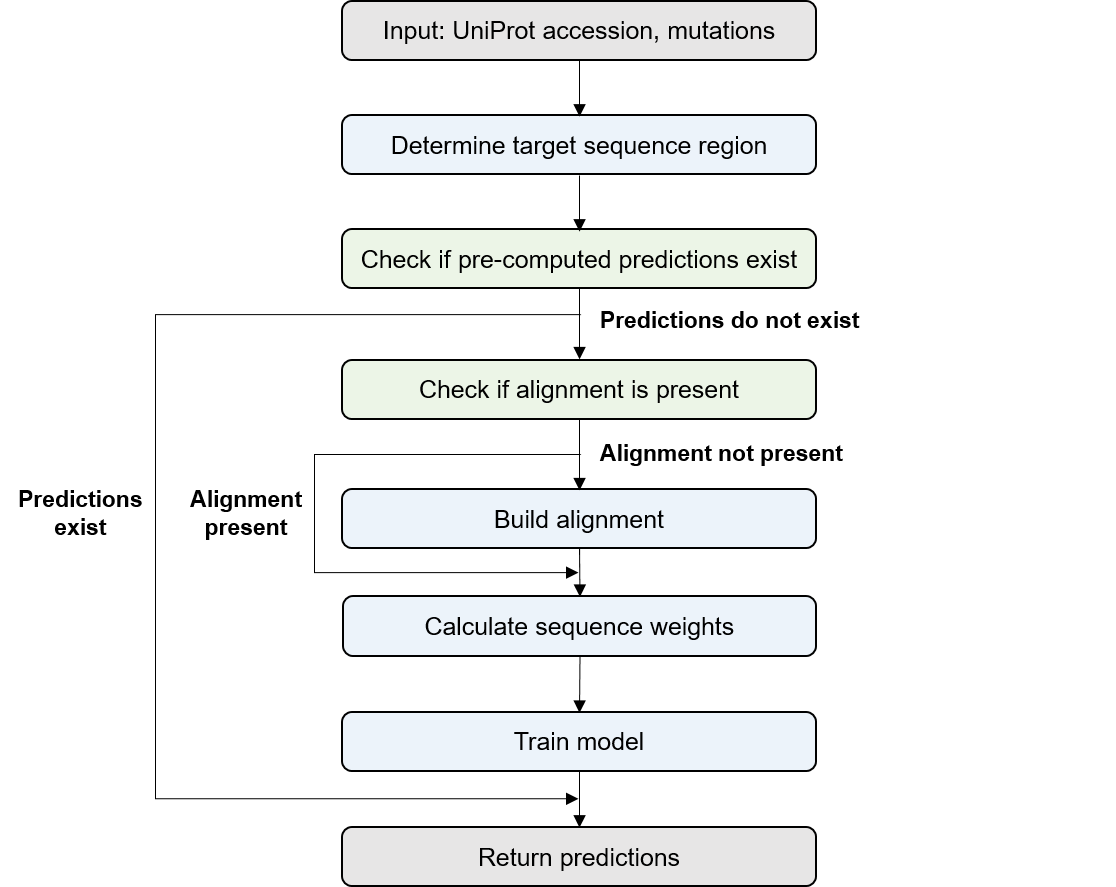

Supplement: Supplementary file 1 — Supplementary Information. [file 41598_2021_98693_MOESM1_ESM.docx]
